# HER2-Low Breast Cancer—Current Knowledge and Future Directions

**DOI:** 10.3390/medicina61040644

**Published:** 2025-04-01

**Authors:** Abeer M. Shaaban, Tanvier Kaur, Elena Provenzano

**Affiliations:** 1 Department of Cellular Pathology, Queen Elizabeth Hospital Birmingham, Birmingham B15 2GW, UK; 2 Cancer and Genomic Sciences, University of Birmingham, Birmingham B15 2TT, UK; 3 Department of Cellular Pathology, New Cross Hospital, Wolverhampton WV10 0QP, UK; tanvier.kaur@nhs.net; 4 Department of Histopathology, Addenbrookes Hospital, Cambridge CB2 0QQ, UK; elena.provenzano@nhs.net; 5 NIHR Cambridge Biomedical Research Centre, Cambridge CB2 0QQ, UK

**Keywords:** HER2-low, HER2-ultralow, T-DXd, breast cancer, artificial intelligence, antibody–drug conjugate

## Abstract

The concept of binary classification of HER2 status has recently been challenged following the DESTINY-Breast trial data showing a clinically meaningful response to antibody–drug conjugates (ADCs) in invasive breast cancer expressing low levels of HER2. HER2-low breast cancer is defined as an immunohistochemistry (IHC) score of 1+ and 2+ without HER2 gene amplification. While HER2-low breast cancer does not represent a biological entity, it encompasses both hormone receptor-positive and triple-negative breast cancer. Differences exist between this group and HER2-null breast cancer. In this review, we provide an update on HER2-low and HER2-ultralow breast cancer, including background trial data, the evolution of HER2-low expression, current clinical guidelines, quality issues, and future directions.

## 1. Introduction

**Figure 1:**
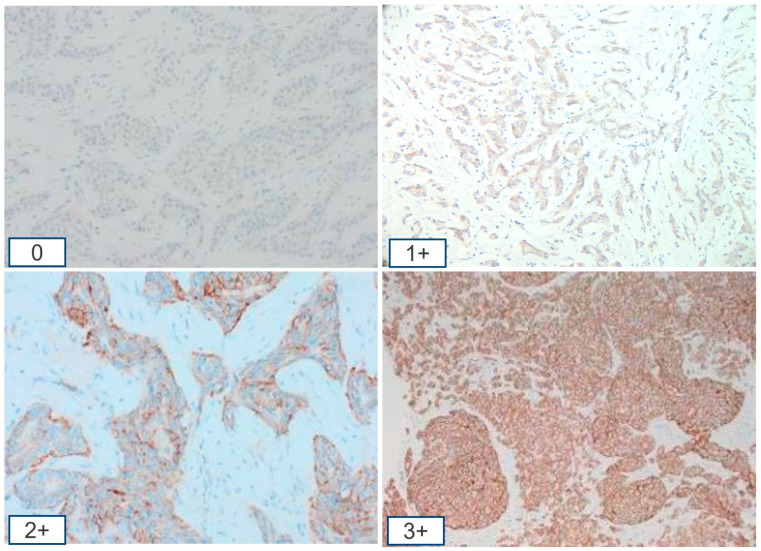
HER2 immunohistochemistry showing the current 4 immunohistochemical categories in breast ductal carcinomas. Scores 1+ and 2+ that are non-amplified by in situ hybridisation are regarded as HER2-low.

Since the identification of Human Epidermal Growth Factor Receptor 2 (HER2) as a target for therapy, the reporting of HER2 expression using a binary system into HER2-positive and -negative has been standard. Immunohistochemistry (IHC) is the main technique and offers a fast, reproducible, and economical method of HER2 protein assessment. Pathologists categorise tumours into four IHC categories based on the intensity of HER2 staining, completeness of membrane positivity, and percentage of positive cells using established guidelines [1,2] (Figure 1). Where IHC is equivocal (score 2+), in situ hybridisation (ISH) techniques such as fluorescence in situ hybridisation (FISH) are used to assess the HER2 gene amplification status. Both IHC and FISH are utilised to identify tumours with HER2 overexpression eligible for standard anti-HER2 therapy. Those comprise IHC score 3+ and HER2 2+ FISH amplified tumours.

The concept of HER2-low breast cancer, which comprises HER2 IHC score 1+ and tumours that are equivocal (IHC 2+) but negative by ISH, has recently been proposed following the publication of the DESTINY trials. Those tumours are currently classified as HER2-negative [3].

A proportion of HER2 IHC score 0 tumours show faint/weak incomplete membrane expression in less than 10% of cells, below the threshold of IHC 1+. Those tumours are categorised as HER2-ultralow [4]. It is important to distinguish them from the HER2-null tumours that show no HER2 membrane staining and are also currently scored as IHC score 0.

Here, we aim to provide an up-to-date review of the current state of knowledge of HER2-low and HER2-ultralow breast cancer, including the biological and clinical, as well as present current challenges in histological diagnosis with recent advances in quantifying HER2 expression such as the use of artificial intelligence and mRNA assays. Using the keywords above, data from the scientific literature in English between 2012 and 2025 were scanned and reviewed.

## 2. Prevalence and Significance

Real-life data show that more than 50% of all breast cancers belong to the HER2-low category. The proportion is higher in the luminal/hormone receptor-positive cancers compared with the triple-negative phenotype [5].

A systematic review of 23 studies comprising 535 patients comparing HER2 scores 0 and 1+ showed that HER2-low breast cancer is more common (67.5%) in the hormone receptor (HR)-positive compared with the HR-negative group (48.6%). While HER2-low breast cancer is not thought to represent a biological entity, an improved disease-free survival was identified in early HER2-low breast cancer regardless of the HR status [6]. Since HER2-low cancer is more prevalent amongst HR-positive cancer, its prognostic significance may be a reflection of this phenotype [7].

While clinical trial data support targeting HER2-low expression in patients, the biological distinction of HER2-low tumours as a separate entity remains unclear. A few studies have suggested that HER2-low tumours possess different genetic profiles compared to HER2-negative tumours, including increased expression of luminal-related genes and lower expression of tyrosine-kinase receptor genes [8,9]. This genetic profile has been further associated with HR-positive status [8]. These findings suggest that HER2-low may represent a quantitative variation in HER2 expression along a biological continuum.

The majority of male breast cancers are HER2-negative. Recent data have shown that they have higher proportions of the HER2-low category compared with their female counterparts. This has no prognostic significance but can be used to guide adjuvant therapy in recurrent/metastatic disease [10].

## 3. HER2-Low Clinical Trials and Antibody–Drug Conjugates (ADCs)

The major trials looking at the role of Trastuzumab deruxtecan (T-DXd) in breast cancer, including HER2-low and now ultra-low disease, are the DESTINY trials (Table 1).

The initial DESTINY01–03 trials established the efficacy of T-DXd in metastatic HER2-positive breast cancer [11,12,13]. Ongoing trials looking at the role of T-DXd in HER2-positive disease include DESTINY05, which compares T-DXd and T-DM1 for patients with early breast cancer who have residual disease post neoadjuvant chemotherapy [14], DESTINY07 and 09 looking at T-DXd in combination with other agents for first-line therapy of metastatic breast cancer [15,16], and DESTINY11 will examine the role of neoadjuvant T-DXd versus standard of care chemotherapy and dual-targeted anti-HER2 therapy for high-risk early breast cancer (T3/4 and/or node-positive) [17].

Based on the efficacy of T-DXd in HER2-positive breast cancer and evidence from pre-clinical models suggesting a clinical effect in tumours with lower levels of HER2 protein expression, a preliminary study by Modi et al. was the first to show a potential clinical role for T-DXd in HER2-low breast cancer [18]. This study of 54 patients with heavily pre-treated HER2-low locally advanced or metastatic breast cancer found an objective response rate (ORR) of 37% with PFS of 11.1 months and overall survival (OS) of 29.4 months. There was no difference in response between IHC 1+ or 2+ breast cancers. This led to the DAISY and DESTINY04 and DESTINY06 clinical trials. The DAISY trial looked at T-DXd in patients with advanced breast cancer divided into 3 cohorts based on HER2 IHC staining in metastatic disease: HER2-positive, HER2-low, and HER2-null [19]. For the HER2-positive group, ORR was 70.6% and PFS was 11.1 months; for the HER2-low group, ORR was 37.5% and PFS was 6.7 months; and for the HER2-0 group, ORR was 29.7% and PFS was 4.2 months. Looking more closely at the HER2-0 cases, those with some HER2 membrane staining (ultra-low) had an ORR of 40%, compared with 25% in those that had no membrane staining (null).

The DESTINY04 trial compared T-DXd with physician’s choice therapy for HER2-low metastatic breast cancer and showed an improvement in PFS and OS for both HR-positive and -negative breast cancers. For the HR-positive group, PFS was 10.1 versus 5.4 months (HR 0.51), OS was 23.9 versus 17.5 months (HR 0.64), and ORR was 52.6% versus 16.3% for T-DXd versus physician’s choice therapy, respectively. For the HR-negative group, PFS was 8.5 versus 2.9 months, OS was 18.2 versus 8.3 months, and ORR was 50% versus 16.7% for T-DXd versus physician’s choice therapy. The findings of the DESTINY04 trial resulted in approval for the use of T-DXd for the treatment of metastatic HER2-low breast cancer in the US and Europe. The DESTINY06 trial explores the benefit of T-DXd in breast cancer with even lower levels of HER2 expression, the ultralow group (weak membrane staining in 0 < x < 10% of tumour cells) [20]. Results show improved PFS (13.2 versus 8.1 months) for T-DXd compared with physician’s choice in this group, with an HR of 0.62. The ORR was 61.8% compared with 26.3%. Overall survival data are still immature, and the final results of this study are eagerly awaited.

**Figure 2:**
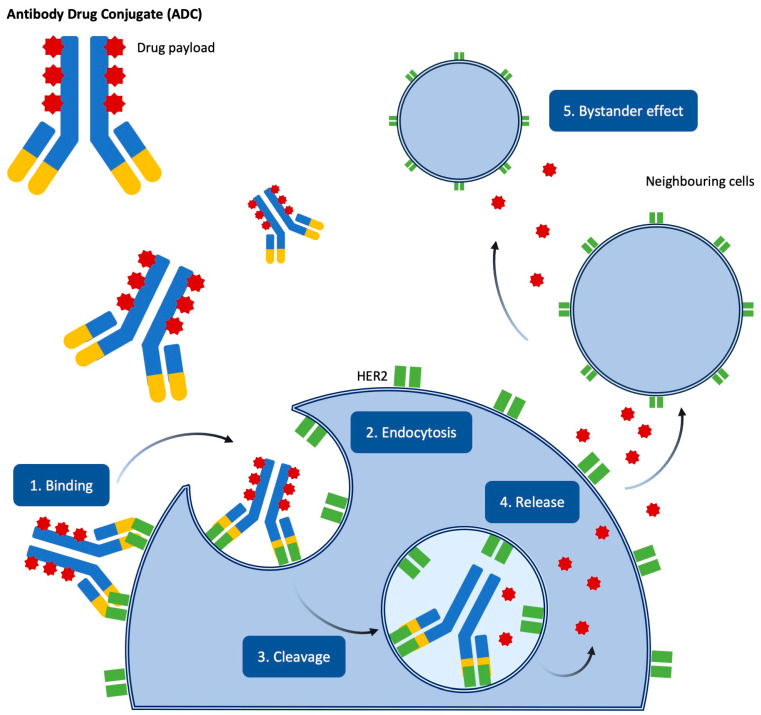
Trastuzumab deruxtecan (T-DXd)—antibody–drug conjugate. T-Dxd binds to HER2 on tumour cells, triggering receptor-mediated endocytosis. Inside the cell, lysosomal proteases cleave the linker, releasing the topoisomerase I inhibitor payload, which induces DNA damage and apoptosis. The membrane-permeable payload also diffuses into neighbouring cells, exerting a bystander effect that enhances the antitumor response.

ADCs were first introduced over 40 years ago, and in 2023, there were 15 ADCs approved by the US Food and Drug Administration (FDA) for use in treating solid tumours and haematological malignancies [21]. ADCs comprise 3 components: a cytotoxic agent, or payload, bound to a monoclonal antibody by a linker molecule (Figure 2). The monoclonal antibody component, most commonly humanised IgG1, provides tissue specificity by binding to a tumour-specific (present on tumour cells only) or tumour-associated (over-expressed by tumour cells relative to normal cells) membrane-bound antigen. IgG1 is preferred because it has a long plasma half-life and high complement activation, resulting in direct and indirect cell killing [22]. The cytotoxic component needs to be a small molecule with very high cytotoxicity, the most common drugs being DNA-damaging agents and tubulin inhibitors [23]. The cytotoxic agent is attached to the monoclonal antibody by a linker molecule that provides drug stability, preventing premature release of the payload before binding the antibody to the target molecule [23]. Upon binding, the ADC is internalised by the tumour cell, resulting in the release of the cytotoxic drug and the monoclonal antibody itself, which can trigger immune cell killing of the tumour cell.

There are two main ADCs that use trastuzumab, a humanised monoclonal antibody directed against HER2, currently licensed for use in breast cancer. HER2 is a membrane-bound receptor overexpressed in 15–20% of breast cancers, although the protein is expressed at low levels on normal breast epithelial cells [19,21].

The first of these is ado-trastuzumab-emtansine (Kadcyla), or T-DM1. After binding to the HER2 receptor, the drug is internalised and broken down in the lysosomes, releasing lysine-MCC-DM1, which disrupts the microtubule network, causing cell death. T-DM1 was approved for use in advanced HER2-positive breast cancer in 2013 based on the results of the EMILIA trial [24], and following the results of the KATHERINE trial, the use has been extended to HER2-positive early breast cancer cases with residual disease post-neoadjuvant chemotherapy [25]. T-DM1 shows higher therapeutic efficiency than trastuzumab and chemotherapy alone for HER2-positive breast cancer, with a manageable side effect profile [26].

The more recent ADC approved for use in breast cancer is fam-trastuzumab Deruxtecan (Enhertu), or T-DXd. T-DXd consists of humanised IgG1 carrying DXd, a potent DNA topoisomerase 1 inhibitor, via an enzymatically cleavable linker. In contrast to lysine-MCC-DM1, DXd is more potent and hydrophobic, allowing tissue permeability with a bystander effect on neighbouring cells. This means that T-DXd is effective in tumours with low or heterogeneous levels of HER2 expression.

There are several other HER2-based ADCs in pre-clinical or early-phase clinical trials, including agents designed to target HER2-low breast cancers [26]. Recently, therapeutic algorithms for the management of HER2-low HR-positive and -negative breast cancer have been proposed [27].

Other ADCs used include sacituzumab govitecan (SG) which is approved for the treatment of metastatic TNBC. The ASCENT trial demonstrated a survival benefit (PFS and OS) with a higher objective response rate in patients with metastatic HER2-low breast cancer who received SG compared with the HER2-negative group [28]. A clinical benefit of using T-DXd following SG was demonstrated [29]. However, a recent multicentre retrospective trial (ADC-low trial) assessing the sequential use of SG and T-DXd in metastatic HER2-low breast cancer revealed limited clinical benefit [30].

Both T-DXd and sacituzumab govitecan are effective agents in the second-line treatment of HER2-low metastatic breast cancer. For resistant hormone receptor-positive HER2-negative breast cancer, patients often receive chemotherapy. Tumours with a HER2-low profile could be treated with either T-DXd or SG, whereas HER2-0 tumours (HER2-ultralow) could be treated with SG [31]. For hormone receptor-negative HER2-low breast cancer after at least one line of chemotherapy, T-DXd or SG should be considered, while HER2-ultralow tumours should be treated with SG followed by other lines of chemotherapy [32,33]. Schlam et al. provided a schema of proposed treatment algorithms for both hormone receptor-positive and triple-negative HER2-low breast cancer [27].

A recent multicentre meta-analysis of 448 patients examined the impact of the HER2-low profile on advanced hormone receptor-positive, HER2-negative breast cancer treated with CDK4/6 inhibitors (CDK4/6i). There was no significant difference in treatment response/PFS between the HER2-low and HER2-null groups [30].

It has recently been shown that endocrine resistance in hormone receptor-positive breast cancer may be linked to the HER2-low status. Several mechanisms are thought to be implicated, including ER pathway hyperactivation, key transcription factor alterations such as *ARID1A*, *MYC*, *CTCF*, *FOXA1*, *TBX3*, MAPK/PI3K-AKT pathway hyperactivation, and subtype switching and phenotypic plasticity in those resistant tumours [34,35]. For a detailed discussion of endocrine resistance mechanisms, refer to Yayli et al., 2024 [36].

## 4. Guidelines

Before the introduction of ADCs, it was only necessary to identify HER2-negative versus HER2-positive breast cancer; the distinction between 0 and 1+ staining had no clinical significance. Following the results of the DESTINY04 trial showing improved survival with ADC therapy even in patients with low levels of HER2 protein expression, the distinction between a 0 or 1+ score, the latter indicating HER2-low status, has become relevant for the management of metastatic breast cancer. In light of this, there have been recent updates in HER2 reporting guidelines for breast cancer.

The updated UK guidelines for HER2 testing were published in December 2022 [1] and focused on several areas, including the concept of HER2-low breast cancer defined as IHC 1+ or 2+ without gene amplification on FISH. More precise definitions of 1+ staining were provided in the hope of improving reproducibility amongst pathologists. HER2 1+ staining is defined as weak, complete membrane staining in <10%, weak incomplete staining in >10%, or faint, barely perceptible staining (complete or incomplete) in >10% of tumour cells. The magnification rule is recommended when assessing staining intensity, with complete staining seen at low power scored as 3+, membrane staining clearly seen at 10x magnification classed as 2+, and staining that is only detectable at 20× or 40× magnification called 1+. The UK guidelines recommend that the actual immunohistochemistry score, with the distinction between 0 and 1+, be included in the histopathology report. The UK guidelines also recommend the introduction of the term HER2-low in histopathology reports for 1+ and 2+ non-amplified breast cancers after approval of the use of ADCs for HER2-low breast cancer has been granted. Routine retesting of tumours with a score of 0 on core biopsy in the surgical excision specimen or following neoadjuvant chemotherapy is not currently recommended, although this decision may need to be reviewed if ADCs become approved for use in the early disease setting.

An update to the US American Society of Clinical Oncology/College of American Pathologists (ASCO/CAP) guidelines for HER2 reporting was published in June 2023 [2]. The authors of the ASCO/CAP update have taken a more cautious approach to the introduction of the new HER2-low terminology. They acknowledge the importance of distinguishing between a score of 0 or 1+ to determine access to T-DXd based on the DESTINY04 trial criteria; however, they state that there is no evidence to support HER2-low as a new or reproducibly defined subtype of breast cancer. The technical difficulties in detecting very low levels of HER2 protein expression are highlighted. The complication for 2+ cases in that HER2-low status is only established when FISH results become available is also mentioned. On these grounds, a decision was made not to alter the current ASCO/CAP HER2 reporting guidelines to incorporate HER2-low status.

Similarly, the European Society of Medical Oncology (ESMO) consensus statements on HER2-low breast cancer were published in June 2023 following consultation amongst a multidisciplinary panel comprising 32 experts [37]. The ESMO statements recommend that pathologists should use the ASCO/CAP 2018 guidelines to determine the HER2 IHC score, and the IHC score (0, 1+, 2+, 3+) should be included in the histopathology report. However, as with the ASCO/CAP update, the ESMO statements do not advocate the use of the term HER2-low in histopathology reports at the current time. It is left to clinicians to determine patient eligibility for T-DXd based on the IHC score provided, i.e., cases with an IHC score of 1+ or 2+ cases that are ISH non-amplified.

It is of note that in the ASCO/CAP guidelines, group 2 cancers (HER2/Cep17 ratio ≥ 2, HER2 copy number < 4) are generally categorised as HER2-negative (HER2-low) [2] but are regarded as HER2-positive in the UK guidelines [1]. A multicentre UK audit of this group showed that they respond to anti-HER2 therapy with a pathological complete response (pCR) rate of 27%, supporting the decision to classify this group as HER2-positive [1].

## 5. Effect of Assay Type

Several immunohistochemical assays are used to analyse HER2 expression in breast cancer. In October 2022, the Ventana Pathway 4B5 assay was approved by the FDA as a companion diagnostic for selecting patients with HER2-low metastatic breast cancer for ENHERTU therapy [38] and in January 2025 for HER2-ultralow for the same indication [39]. The UK, US, and ESMO guidelines [1,2,37] address the issue of which diagnostic test should be used to determine HER2 status. Although the DESTINY04 and 06 trials used the Ventana Pathway HER2 (4B5) test [20,33], the guidelines all state that any validated HER2 assay may be used, provided appropriate quality assurance measures and external proficiency testing is adhered to.

It is of note that the proportion of HER2-low breast cancer depends on the type of immunohistochemical assay/antibody used, and some tumours will be categorised differently based on the assay used [40]. A study of 119 breast cancers using the two most widely used assays (HercepTest™and Ventana Pathway 4B5 assays) showed a concordance rate of 98.2% in classifying tumours as HER2-positive versus -negative. More cases were classified as HER2-low (35%) by the former compared with the latter (19%), with more cases classified as HER2 2+ using the Hercep Test compared with the Pathway 4B5 assay (27% vs. 15%, respectively) [41]. A recent multicentre Dutch study of 35 laboratories using three slides with dynamic range cell lines and IHC analyte conjugated microbead calibrators compared 4 different routine HER2 antibodies: 4B5, A0485, DG44 (HercepTest), and SP3 [42]. For the 4B5 assay, two different detection kits were used including the ultraView and OptiView. AI analysis showed interlaboratory variation in HER2 cell line expression. Overall, the calibrators DG44 and 4B5 OptiView had the highest, almost identical, analytical sensitivity, followed closely by 4B5 ultraView, while SP3 was the least sensitive. It is of note that the DB-04 trial used the 4B5 antibody with ultraView detection [33]. Both the 4B5 and HercepTest are ready-to-use (RTU) kits, minimising interlaboratory variation for all cores.

In addition, data from the first 7 assessment runs of the UKNEQAS HER2-low scheme showed significant variation in the quality of HER2 immunohistochemical staining, with over 50% of laboratories failing to detect the lower levels of expression [43]. The Ventana 4B5 clone was the most commonly used primary antibody (87.3%), with a pass rate of 70.3%. Only 5.6% of the submitting laboratories used the CB11 clone (Oracle, Leica), none of which achieved a pass. The above data highlight that various pre-analytical and analytical factors can lead to inter-laboratory variability in assay testing with consequent differences in the population of patients selected for ADC therapy and for clinical trials.

One important question is whether the current assays designed to detect HER2 overexpression are suitable for selecting tumours with low expression levels. Other technologies, such as mRNA-based tests, have been developed and have shown promising results in quantifying the amount of EGFR2 mRNA and selecting the HER2-low group [44], but these are not yet implemented in routine practice.

## 6. Quality Assurance

Detection of low levels of HER2 staining requires the use of quality-assured assays with appropriate controls to ensure consistency and reproducibility of staining. Mandatory cell line controls, preferably on the same slide as the test section, should be included. The latter can be cell line controls, preferably on the same slide as the test section. UKNEQAS currently recommends the use of controls spanning the whole spectrum of staining scores (0, 1+, 2+, 3+). Additionally, inter-lab calibration protocols should be implemented to ensure consistent interpretation across laboratories.

Pre-analytical causes, such as specimen fixation, have been shown to affect the interpretation of HER2 expression in core and surgical excision samples [45], and care should be given to minimise cold ischaemic time due to delays in fixation.

A number of HER2 quality assurance and HER2 educational schemes are available for pathologists and laboratories worldwide. Examples of those include the UK NEQAS UK NEQAS ICC & ISH Modules, NordiQC in Denmark NordiQC—Immunohistochemical Quality Control, QuIP in Germany Quality in Pathology—QuIP: Home, CADQAS in the UK CADQAS—Cancer Diagnostic Quality Assurance Services—Testing and, more recently, a freely available educational HER2-low portal including training and testing slide sets HER2-Low Training Module—Overview. These will allow regular proficiency testing and educational support for Her2-low identification.

## 7. Evolution of HER2-Low Expression

Consideration of tumour sample selection is important in view of the general availability of more than one tumour sample per patient, including diagnostic core biopsy, surgical excisions, treatment naïve and post-treatment tumours, nodal and distant metastases, etc. This begs the question of whether HER2-low expression is static or undergoes evolution in various stages of the disease.

Recent data have shown a considerable difference in HER2-low status between core and surgical excisions. In a paired analysis of 5610 core and untreated breast cancer excision samples, Lu and colleagues reported an overall concordance of only 76.78%. A total of 530/1066 HER2-null cancers were reclassified as HER2-low, highlighting the importance of retesting another sample for HER2-null tumours [46]. In another paired analysis of 57 core and excision samples of HER2-negative breast cancer, 49% and 44% of the cores and excisions were classified as HER2-low, respectively [47].

Miglietta et al. reported a slightly increased overall rate of HER2-low cancer in the metastases compared to primary cancers (37.3% vs. 34.2%). The overall rate of HER2 discordance between primary and secondary carcinoma was 38.0%, with HER2-null tumours switching to HER2-low (15%) and HER2-low cancers losing expression to become HER2-0 (14%) [48]. In a study of 98 HER2-low primary breast cancers confirmed on review, 22.4% switched to a score of 0 in metastasis. Interestingly, a significantly higher rate of discordance was found with increasing T stage and was influenced by the site of metastasis. The highest discordance was seen in bone metastases, which should, therefore, be avoided where possible, whereas the lowest occurred in lung metastases [20].

Previous literature has shown a change in HER2 status from positive to negative (more common) and from negative to positive following neoadjuvant chemotherapy (NACT) [49,50]. In the HER2-low setting, a 32% discordance between pre- and post-NACT tumour samples was reported with a switch from score 0 to 1+ and vice versa. Following NACT, the rate of pCR was significantly lower (4% versus 5%; *p* = 0.022) in the HER2-low group [51]. Furthermore, short-course (bridging) endocrine therapy resulted in a significant change in HER2-low status, with 32% of cases switching to a HER2-0 profile following NAET [52].

## 8. Concordance of Pathologists Scoring of HER2-Low Breast Cancer

The concept of HER2-low breast cancer has intensified the focus on reproducibility of pathologist scoring of HER2. An early study of 127 cases reviewed by 3 pathologists found the best agreement was for 0 and 3+ cases, and the worst was 1+ and 2+ [49]. The main sources of discordance were heterogeneity, more common in HER2-low breast cancer, and interpretation of non-specific staining [53].

Several recent studies have analysed HER2-low and HER2-ultralow diagnostic reproducibility. Zaakouk et al. reported low concordance among 16 expert pathologists from the UK and Ireland in differentiating HER2-low and -ultralow cancers in a series of 50 breast cancers enriched and scored using digital whole slide images (WSI). Reasons for discordance include heterogeneity of HER2 expression, background cytoplasmic staining, and difficulties in assessing percentage expression at the cut-off value of 10%. The highest concordance (86%) was achieved by clustering any positivity (score 1+, 2+, 3+) against score 0 [54]. Indeed, it was suggested that grouping any percentage of positivity versus the null group may be the way forward to ensure consistency and inform eligibility for ADCs [54].

A recent study of 36 pathologists examining 50 HER2-stained cancers, all previously reported as HER2 IHC 0, demonstrated poor concordance in categorising HER2-null and HER2-ultralow with a Fleiss κ score of only 0.230 [55].

Fernandez et al. examined the results of the College of American Pathologists proficiency testing over a 2-year period (2019/2020) involving 1391 and 1452 laboratories, respectively [56]. Two-thirds of cases showed 90% or more agreement, all of which were 0 or 3+. The lowest agreement was for the distinction of 0 versus 1+, where a quarter showed less than 70% concordance.

In a review of 170 core biopsies by 18 pathologists from 15 centres, 92 cases were scored as 0 by at least one pathologist, with only 26% having more than 90% agreement, in contrast to 45 cases scored as 3+, where 58% showed 90% or more agreement [57]. Of 102 cases scored as 1+, only 1 case showed complete agreement across pathologists [57]. When cases were considered as 0 versus not 0, the OPA was 59.4%, compared with 87.1% for 3+ versus not 3+. The authors concluded that current immunohistochemical tests perform well for identifying high levels of expression, as they were designed to do, but perform less reproducibly in discriminating low levels of protein expression, as in HER2-low.

A large international ring study of 105 HER2-negative core biopsies scored by 16 specialist breast pathologists found interobserver agreement of 0.63 with complete agreement in 4.7% of cases; when 1+ and 2+ scores were combined, this improved to complete agreement in 33% of cases [58]. A consensus meeting identified areas of difficulty, including the presence of non-specific staining, the definition of ‘barely perceptible’ in the updated 2018 guidelines, and cases around the 10% cut-off. A second round of scoring was performed, including the ‘ultralow’ category with interobserver agreement of 0.32; however, when 0 versus any staining was considered, there was complete agreement in 74% of cases.

A review of data from the Danish breast cancer clinical database of over 48,000 patients tested in 14 laboratories found large variations in the rates of 0, 1+, and 2+ cases between laboratories and across the time period [59]. The proportion of cases scored 0 ranged from 10.7 to 38.1%, 1+ from 35.8 to 58.8%, and 2+ from 6.7 to 31.0%, compared with 9.5 to 15.6% for 3+ cases. In contrast, positivity for oestrogen receptors ranged from 81.8% to 88.2%. Over the time period of the study, the HER2-low rate ranged from 49.3% to 65.6%, compared with 12.6% to 15.7% for HER2-positive breast cancer. Over the most recent 6 years, testing was performed in 12 labs, 11 of which were using the 4B5 antibody on the Ventana platform, suggesting this variation is largely related to differences in interpretation rather than staining.

The Australian HER2-Low Breast Cancer Concordance Study, comprising 9 expert breast pathologists, analysed WSI of 60 HER2 stained tumours stained by 4B5 assay from 3 lobotomies and mainly originally scored as IHC 0 or 1+. Tumours with HER2 staining in less than 20% of cells had the lowest concordance. Out of 17 cases locally reported as HER2-0, 7 (41.25) were reclassified as IHC 1+, whereas 7/32 cases (21.8%) locally scored as IHC 1+ were scored by experts as IHC 0 (HER2-ultralow or -null). Individual pathologists’ concordance with the consensus score ranged from 73.3% to 91.67% [60].

The value of education and training has been emphasised in a recent study of 77 pathologists from 14 countries. A virtual four-hour education session increased the concordance of the HER2-low category (using the 4B5 assay) from 80.6% to 91.1% after training (*p* < 0.001) [61].

## 9. Artificial Intelligence

The introduction of digital pathology, whereby glass histology slides are scanned to generate whole slide images (WSI), which are then viewed on a computer screen rather than with a microscope, is transforming the way pathology is practised. The generation of digital WSI also paves the way for the use of artificial intelligence (AI) in the interpretation of histological findings and scoring immunohistochemical markers such as HER2, including going beyond what the human eye can perceive. Interobserver reproducibility in the scoring of HER2, especially the HER2-low subgroup, is suboptimal, as described previously. The use of AI algorithms to assist pathologists in scoring HER2 is one potential solution [62,63].

**Figure 3:**
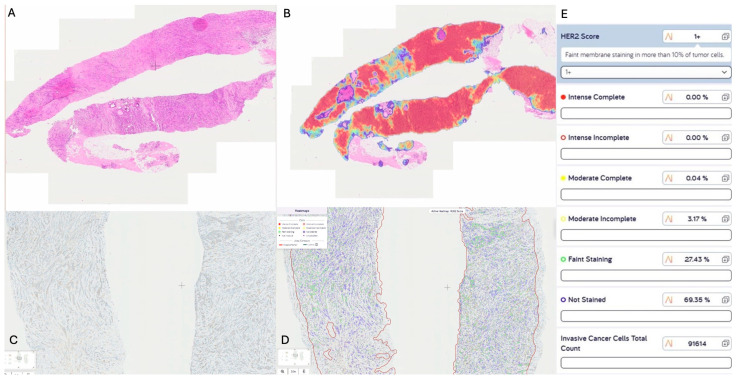
Pathway for AI analysis of HER2-stained slide. (**A**). H&E stained slide of core biopsy with invasive cancer. (**B**). Heat map with area of invasive cancer identified. (**C**). Digital HER2-stained slide. (**D**). HER2 membrane staining pattern and intensity quantified for each invasive tumour cell. Blue circles are cells with no staining. Green circles are cells with faint membrane staining. Yellow circles are cells with moderate incomplete membrane staining. E. Overall HER2 score based on percentage of cells with each staining intensity.

There are several commercial AI algorithms for scoring HER2 that are currently available for analysis of HER2-stained WSI. Visiopharm HER2-CONNECT, Paige HER2Complete, and Ventana uPath HER2 are all CE-marked, and the IBEX Galen Breast HER2 is in development [64]. The first step in the algorithm is the identification of invasive tumour cells, which is the relevant cell population to be scored. The algorithm then determines the intensity (strong, moderate, weak) and pattern of staining (circumferential versus partial) around each invasive cancer cell to provide a breakdown of the percentage of cells in each HER2 category—0, 1+, 2+, 3+ —and on the basis of these percentages assigns an overall HER2 score using the ASCO/CAP criteria (Figure 3). There are published guidelines for laboratories for the incorporation of HER2 quantitative image analysis systems into routine practice, including training, validation, and ongoing monitoring of results [65].

Studies looking at AI- based scoring of HER2 have shown improvement in interobserver reproducibility and accuracy in scoring when compared with ground truth scores as determined by expert pathologists, especially within the HER2-low subgroup. A recent literature review and meta-analysis identified 13 studies suitable for inclusion, although only 4 examined WSI rather than selected patches or regions of interest, and 9 reported AI-only rather than pathologist-assisted results. Overall, the sensitivity and specificity for score 0 were 0.97 and 0.82, 1+ was 0.69 and 0.94, 2+ was 0.89 and 0.96, and 3+ was 0.97 and 0.99. Therefore, as with pathologist reproducibility, AI shows the greatest reliability for 0 and 3+ results, although this may also reflect the limitations of using pathologist scoring as the gold standard [66].

Intratumoural heterogeneity in HER2 expression is an important source of reduced reproducibility in scoring of HER2. Pathologists find cases with variability in intensity and completeness of staining challenging to interpret, and intratumoural heterogeneity is more commonly encountered in cases with 1+ and 2+ HER2 staining, i.e., HER2-low cancers, with up to 86% of 1+ cases showing variable expression of HER2 protein [67].

In one study, AI assistance improved the accuracy in the scoring of 1+ cases from 0.70 to 0.90, and in cases with heterogeneity, accuracy improved from 0.68 to 0.92. Another recent study of a commercial algorithm showed that the accuracy of the AI alone was 93% of 0, 90% of 1+, 87.5% of 2+, and 100% of 3+ cases. There was a significant improvement in pathologist agreement with ground truth scores for 0 (75% to 92.5%), 1+ (62% to 72.5%), and 3+ (94% to 97%) cases, but interestingly not for 2+ (63% to 54%) [68]. An extended study using the same algorithm involving more centres has shown a similar improvement in accuracy and a reduction in the 2+ rate, which could result in reduced FISH testing with cost savings and improved turnaround times.

Beyond quantitative image analysis to improve the reproducibility of scoring HER2 IHC, there have been some interesting studies exploring the potential of AI to predict HER2 status based on the H&E tumour characteristics. So far, these have shown some promise in identifying HER2-positive cases but are less able to identify HER2-low cancers, possibly reflecting the fact that these do not seem to represent a distinct biological entity. Gustavson et al. used a deep learning approach to develop an AI-derived HER2 Quantitative Continuous Score (QCS) that predicted response to T-DXd in HER2-low cancers; the QCS high group had 53% response with a median PFS of 14.5 months, whilst the QCS low group 24% responded with median PFS of 8.6 months [69]. The QCS cut-off and a separate spatial prediction score were driven by the majority of cells expressing minimal levels of protein rather than a small proportion of cells with higher protein expression [69]. Another interesting study used AI to look at the intensity and pattern of HER2 expression in metastatic core biopsies and correlated it with T-DXd uptake and response in the DAISY trial [19]. High levels of HER2 protein expression correlated with T-DXd uptake into cells, whilst HER2-0 cases showed no or very few T-DXd staining. However, two of the three HER2-0 cases with low T-DXd distribution had a partial response to therapy. In cases that developed resistance, HER2 expression decreased.

It is important to remember that whilst these AI tools will help with reproducibility in terms of scoring the slides, the end result is still dependent on the quality of the initial staining and the scanned WSI. Pre-analytical factors such as antibody clone, staining methodology, and scanning systems can introduce subtle discrepancies in colour and image quality, which, in turn, may impact the accuracy of AI-based scoring systems [70]. Additionally, variability between different scanning systems, including differences in resolution, colour calibration, and image compression, can further affect the consistency of digital slides, posing additional challenges for AI algorithms [63]. These factors highlight the importance of standardisation across both pre-analytical processes and scanner calibration to ensure the reliability of AI-based tools in clinical practice.

## 10. Future Directions

The current availability of ADCs opens a new horizon into the treatment of a large number of breast cancer patients with metastatic disease. Algorithms have been developed for adequate management of recurrent and metastatic HER2-low and -ultralow breast tumours of either luminal or triple-negative phenotype. However, accurate patient selection is paramount for optimising treatment outcomes. Further work on refining the HER2-low and -ultralow definition, such as using dynamic range assay and IHC calibrator spots, taking into account heterogeneity of HER2 expression, is needed and will be further informed by final data from the DESTINY- Breast 06 trial. The role of AI and mRNA-based technologies is evolving, but their application in clinical practice still requires validation. Establishing consistent protocols for mRNA extraction, quantification, and analysis is essential for accurate patient stratification. Variations in these processes can lead to discrepancies in results, complicating the reliable classification of tumours as HER2-low or HER2-ultralow, particularly as the definition of HER2-ultralow continues to be refined. Therefore, prioritising research to standardise these methods is crucial to ensure accurate HER2 assessment and support the ongoing efforts to define HER2-ultralow status.

Furthermore, looking into the future of HER2 assessment in breast cancer, it will be essential to carefully evaluate the integration of mRNA-based assays with existing methodologies, such as immunohistochemistry and fluorescence in situ hybridisation, to establish the most effective diagnostic approach.

Finally, continued liaison between members of the multidisciplinary teams and ongoing education and training are required to deliver optimised, individualised patient management.

## Figures and Tables

**Table 1:** Summary of DESTINY trials.

Trial	Study Population	Phase	Treatment Arms	Primary Endpoint	Outcome
DESTINY04	Metastatic HER2-low	3	T-DXd vs. physician choice	PFS * in HR-positive cohort	Improved survival with T-Dxd
DESTINY06	Metastatic HER2-low and HER2-ultralow	3	T-DXd vs. physician choice	PFS in HER2-low breast cancer. Secondary endpoint PFS in all patients	Improved survival with T-DXd in both groups
DAISY	Locally advanced/metastatic -HER2-positive/HER2-low/ HER2-null	2	Single arm T-DXd	Objective response rate in the 3 HER2 cohorts	Primary endpoint met in cohorts 1 and 2—70.6% cohort 1 and 37.5% cohort 2

* PFS = Progression Free Survival.

## Data Availability

No new data were created or analysed in this study.
